# A novel index equivalent to the myocardial performance index for right ventricular functional assessment in children and adolescent patients

**DOI:** 10.1038/s41598-019-56564-y

**Published:** 2019-12-27

**Authors:** Yasunobu Hayabuchi, Yukako Homma, Shoji Kagami

**Affiliations:** Department of Pediatrics, Tokushima University, Tokushima, Japan

**Keywords:** Cardiology, Paediatric research

## Abstract

The aims of the present study were to develop and check the utility and feasibility of a novel right ventricular (RV) functional index (RV angular velocity; RVω, s^−1^) derived from the angular velocity in harmonic oscillator kinematics obtained from the RV pressure waveform. We hypothesized that RVω reflects the myocardial performance index (MPI), which represents global RV function. A total of 132 consecutive patients, ranging in age from 3 months to 34 years with various cardiac diseases were included in this prospective study. RVω was defined as the difference between the peak derivative of pressure (dP/dt_max − dP/dt_min) divided by the difference between the maximum and minimum pressure (Pmax – Pmin). RVω showed significant negative correlations with the pulsed-wave Doppler-derived myocardial performance index (PWD-MPI) and the tissue Doppler imaging-derived MPI (TDI-MPI) (r = −0.52 and −0.51, respectively; both p < 0.0001). RVω also showed significant positive correlations with RV fractional area change (RVFAC) and RV ejection fraction (RVEF) (r = 0.41 and 0.39, respectively; both p < 0.0001), as well as a significant negative correlation with tricuspid E/e′ (r = −0.19, p = 0.0283). The clinical feasibility and utility of RVω for assessing global RV performance, incorporating both systolic and diastolic function, were demonstrated.

## Introduction

The myocardial performance index (MPI), also known as the Tei index, is an assessment of global myocardial function that incorporates both systolic and diastolic performance and can be applied to the left ventricle (LV) and right ventricle (RV)^1–6^. Many clinical investigations have demonstrated the diagnostic and prognostic reliability of the MPI in a wide variety of congenital and acquired cardiovascular abnormalities^2,5–8^. The RV MPI has been reported to correlate with clinical status and predict outcome in patients with idiopathic pulmonary arterial hypertension or with congenital heart diseases^5,6,8–10^. However, there are several important technical and conceptual limitations in the assessment of the MPI. The precise onset and endpoints of pulsed Doppler wave tracings may be obscure, particularly at low blood flow or tissue velocities. This would impair time-interval measurements in clinical practice, with potential unsatisfactory accuracy of MPI measures^11–13^. Furthermore, it has been reported that the RV MPI can show pseudonormalization in patients with severe RV dysfunction due to RV infarction or in patients with surgically repaired tetralogy of Fallot^14,15^. In addition, the effects of valve dysfunction on the MPI must also be considered when evaluating ventricular function in patients with valve disease^16,17^.

Cardiac function analysis consists of volume analysis, pressure analysis, and time-phase analysis. The MPI is the index that evaluates overall cardiac function from the viewpoint of time-phase analysis using echocardiography, whereas there are no time-phase analysis parameters obtained from cardiac catheterization that correspond to the MPI. Furthermore, there is no cardiac catheterization-derived ventricular functional index that incorporates both systolic and diastolic functions. In the present study, a novel index derived from time-phase analysis using the RV pressure waveform was developed, and its utility and feasibility in paediatric patients were examined.

## Methods

### Study design and patient population

The subjects in this prospective investigation were 132 consecutive paediatric patients (mean age ± standard deviation [SD], 6.9 ± 9.0 years; age range, 3 months – 34 years). All participants had been scheduled for their circulatory evaluations. To test the generality of the novel RV pressure waveform-derived index, the patients were chosen to be clinically heterogeneous. The patients’ diagnoses and characteristics are summarized in Table 1.

**Table 1:** Clinical characteristics of the participants (n = 132).

Sex (male/female)	56/76
Age	6.9 ± 5.1 years (3 months – 34 years)
Weight (kg)	21.3 ± 20.8 (4.0–83.0)
Height (cm)	97.7 ± 37.8 (53.0–176.5)
Body surface area (m^2^)	0.72 ± 0.49 (0.32–1.67)
**Cardiac diseases**	
Preoperative VSD	24
Postoperative VSD	11
Preoperative ASD	8
PDA	10
VSD after PAB	10
TOF	2
Postoperative TOF	22
Postoperative TGA	9
Postoperative CAVSD	7
Postoperative CoA	3
Preoperative PAPVC	1
MSR	3
Postoperative TAPVC	1
Postoperative TAC	1
PS	2
ASR	1
Postoperative PAIVS	2
Postoperative ALCAPA	1
PAH	10
DCM	1
RCM	1
KD	1
Pulmonary sequestration	1
Heart rate (bpm)	91.5 ± 20.0 (51–126)
LVEF (%)	71.8 ± 7.5 (45–81)
RVFAC (%)	36.3 ± 11.1 (16.0–63.0)
RVEF (%)	57.4 ± 9.1 (34.0–74.0)
LVSP (mmHg)	74.7 ± 12.7 (49–122)
LVEDP (mmHg)	10.3 ± 4.6 (2–23)
LV time constant (τ)	31.3 ± 13.6 (12.0–94.2)
RVSP (mmHg)	37.5 ± 19.0 (10.0–90.5)
RVEDP (mmHg)	5.2 ± 3.8 (0–15)
RV time constant (τ)	33.3 ± 14.7 (7.6–79.0)

ALCAPA, anomalous origin of the left coronary artery from the pulmonary artery; ASD, atrial septal defect; ASR, aortic stenosis and regurgitation; CAVSD, complete atrioventricular septal defect; CoA, coarctation of the aorta; DCM, dilated cardiomyopathy; KD, Kawasaki disease; LVEDP, left ventricular end-diastolic pressure; LVEF, left ventricular ejection fraction; LVSP, left ventricular systolic pressure; MSR, mitral stenosis and regurgitation; PAB, pulmonary artery banding; PAH, pulmonary arterial hypertension; PAIVS, pulmonary atresia with intact ventricular septum;

PAPVC, partial anomalous pulmonary venous connection; PDA, patent ductus arteriosus; PS, pulmonary stenosis; RCM, restrictive cardiomyopathy; RVEDP, right ventricular end-diastolic pressure; RVEF, right ventricular ejection fraction; RVFAC, right ventricular fractional area change; RVSP, right ventricular systolic pressure; TAPVC, total anomalous pulmonary venous connection; TAC, Truncus arteriosus communis; TGA, transposition of the great arteries; TOF, tetralogy of Fallot; VSD, ventricular septal defect. Values are presented as means ± SD; the range for each variable is indicated in parentheses.

Data acquired from December 2014 to October 2018 were analysed. All study protocols were approved by the Institutional Review Board of the Tokushima University Hospital with written informed consent provided by the patients or their parents, and was conducted in accordance with the provisions of the Declaration of Helsinki.

### Echocardiographic study

Standard echocardiographic examination was performed using a Preirus digital ultrasound system (Hitachi-Aloka Medical Co., Tokyo, Japan). All data were obtained from patients in the left lateral decubitus position during end-expiratory apnoea.

**Figure 1:**
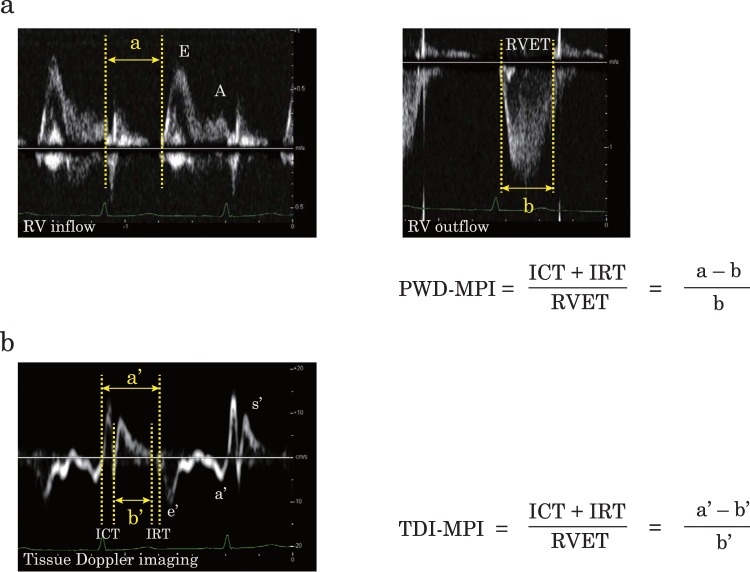
Measurement of PWD-MPI and TDI-MPI. The pulsed-wave Doppler (PWD) (**a**) and tissue Doppler imaging (TDI) (**b**) was recorded from 14-year-old surgically repaired tetralogy of Fallot. The time interval “a” was measured between cessation and onset of RV inflow. The time interval “b” was measured between onset and cessation of RV outflow. PWD-MPI was calculated as shown (**a**). The time interval “a’” was measured between the end and onset of tricuspid annular diastolic velocities. The time interval “b’” was measured the duration of tricuspid annular systolic velocity. TDI-MPI was calculated as shown (**b**). ICT, isovolumic contraction time; IRT; isovolumic relaxation time; PWD-MPI, myocardial performance index evaluated using pulsed-wave Doppler; RVET, right ventricular ejection time; TDI-MPI, myocardial performance index evaluated using tissue Doppler imaging.

Tricuspid inflow velocities were recorded from the apical four-chamber view with the pulsed-wave Doppler (PWD) sample volume placed at the tips of the tricuspid leaflets. The time interval shown as “a” was measured between cessation and onset of the tricuspid inflow (Fig. 1a). The pulsed Doppler recording of RV outflow was made placing the sample just below the pulmonary valve. The interval “b” shows the ejection time, and was determined between onset and end of RV outflow. PWD-MPI was calculated by the following formula: PWD-MPI = (a − b)/b^1–3^.

Tissue Doppler imaging (TDI) was performed from the apical four-chamber image by placing the Doppler sample volume on the tricuspid lateral annulus. The TDI-MPI was measured using the following formula: TDI-MPI = (a′ − b′)/b′, where a′ is the interval between cessation and onset of tricuspid annular diastolic velocities, and b′ is the duration of tricuspid annular systolic motion (Fig. 1b)^18,19^.

In addition to measuring the MPI, patients were evaluated by conventional echocardiography as follows. The LV ejection fraction (LVEF) was measured from apical two-chamber and four-chamber views using the biplane Simpson’s technique. RV fractional area change (RVFAC) was evaluated from the apical four-chamber view. All measurements were performed over three cardiac cycles and then averaged.

### Cardiac catheterization

All participants underwent cardiac catheterization within 3 days of echocardiographic examination. RV pressure (RVP) measurement was acquired using a high-fidelity manometer-tipped 0.014-inch pressure wire (PressureWire Aeris; Abbott Vascular Japan co. ltd, Tokyo, Japan). All pressure data were obtained at a sampling rate of 100 Hz during respiration suspended at the end of expiration.

### Novel parameter obtained from the pressure-phase plane (PPP)

**Figure 2:**
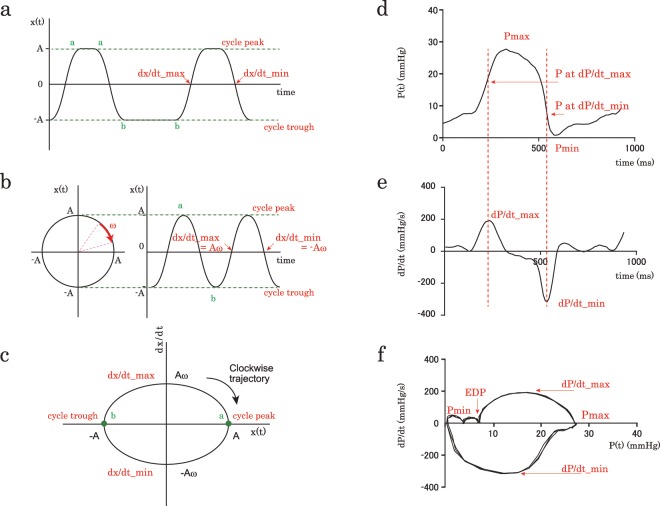
A novel index, RVω, obtained from the right ventricular pressure contour. The waveform mimicking right ventricular pressure is shown (**a**). The simple harmonic oscillation/sine curve (**b**) created by eliminating the interval a-a (ejection time) and b-b (diastolic duration) from the (**a**) waveform. The diagrammatic representation of angular velocity (ω; radians/s) is shown on the uniform circular motion display. The kinematic phase plane (dx/dt vs. x) of the harmonic oscillator (**c**) can be the same for the waveforms of (**a**,**b**). The angular velocity (ω) is expressed by the ratio of the maximum y-axis position of the loop to half the width on the x-axis (Aω/A). Representative examples of the time courses of right ventricular pressure (**d**) and the time derivative of pressure (dP/dt) vs. time data (**e**) sets are shown. The pressure-phase plane (PPP; by replacing displacement x with pressure P) also traces a clockwise path, reflecting events of the cardiac cycle (**f**). The PPP-derived algebraic expression for the angular velocity (RVω) is applied to the novel analogous index, which is calculated as (dP/dt_max − dP/dt_min)/Pmax- Pmin. ω, angular velocity; Pmax, maximum right ventricular pressure; Pmin, minimum right ventricular pressure; dP/dt_max, maximum time derivative of right ventricular pressure; dP/dt_min, minimum time derivative of right ventricular pressure; EDP, right ventricular end-diastolic pressure; τ, time constant of right ventricular relaxation.

The waveform mimicking RVP is shown in Fig. 2a. The intervals a-a and b-b in Fig. 2a correspond to ejection time and diastolic duration depending on the cardiac cycle time, respectively. Figure 2b shows the simple harmonic oscillation/sine curve that is formed by eliminating the interval a-a and b-b from the Fig. 2a waveform. The diagrammatic representation of angular velocity (ω; radians/s) in the simple harmonic oscillation/sine curve is added by the uniform circular motion display on the left side of Fig. 2b. The kinematic phase plane for a simple harmonic oscillator plots velocity (dx/dt) vs. position [x(t)] of the harmonic oscillator (Fig. 2c). The kinematic phase plane of waveforms of Fig. 2a,b can be the same and is shown as Fig. 2c, because the time intervals a-a and b-b do not have an effect on the phase plane configuration. Then, the kinematics were applied to the RVP waveform. RVP data [P(t)] were converted to digital data (Fig. 2d), and the time derivative of pressure (dP/dt) vs. time data sets were digitally smoothed using a five-point average to suppress small noise (Fig. 2e). As shown in Fig. 2f, the loop in the pressure-phase plane (PPP; by replacing displacement x with pressure P) also traces a clockwise path, reflecting events of the cardiac cycle^20,21^. The PPP configuration is not affected by the heart rate, but analogously determined by the amplitude of Pmax –Pmin, and the angular velocity of ω, which affects dP/dt_max and dP/dt_min.

The kinematic phase plane-derived algebraic expression for the angular velocity (ω) was applied to the novel analogous index that quantifies the time-phase index, MPI. We postulated that the PPP-derived index, angular velocity of RVP (RVω), can be an alternative to the RV MPI.

### Determination of angular velocity (ω) from the PPP

The kinematics of the phase plane is governed by the (mass-normalized) equation of motion as follows:

1 $$\frac{{{\rm{d}}}^{2}{x}}{{\rm{d}}{{t}}^{2}}+c\frac{{\rm{d}}{x}}{{\rm{d}}t}+kx=0$$

where c is the damping coefficient, and k is the spring constant^22^. Both c and k are mass-normalized (per gram) constants^22^.

An approximate method was proposed for deriving angular velocity (ω) from the geometric characteristics of the PPP loop generated by the RVP. Assuming undamped (c = 0) oscillation (because the simple harmonic oscillation/sine curve is assumed), the solution to Eq. 1 is as follows:

2 $$x=A\cdot \,\cos ({\rm{\omega }}\cdot t)$$

and

3 $$\frac{{\rm{d}}x}{{\rm{d}}t}=-\,A{\rm{\omega }}\cdot \,\sin ({\rm{\omega }}\cdot t)$$

where A is the initial displacement, and ω is the angular velocity given by the mass-normalized spring constant k (ω = k^1/2^). The ellipse-shaped kinematic phase plane loop traces a clockwise trajectory (Fig. 2c).

The intercept of the loop on the velocity dx/dt and displacement x-axis can be related to the angular velocity via the following relation:

4 $${\rm{\omega }}=\frac{A{\rm{\omega }}}{A}$$

Therefore, the angular velocity (ω) can be calculated from the ratio of the maximum y-axis of the loop (Aω) to half the width on the x-axis (A). Therefore, by replacing x and dx/dt with P and dP/dt, RV angular velocity (RVω) can be defined as the difference between the peak derivative of pressure (dP/dt_max − dP/dt_min) divided by the difference between the maximum and minimum pressures (Pmax – Pmin) or:

5 $${\rm{RV}}{\rm{\omega }}=\frac{{\rm{dP}}/{\rm{dt}}\_\,{\rm{\max }}\,-{\rm{dP}}/{\rm{dt}}\_\,{\rm{\min }}}{{\rm{Pmax}}-{\rm{Pmin}}}$$

A PPP-derived index for angular velocity can then be obtained by using the analogous features of the loop inscribed by the RVP contour.

### Statistical analysis

All results are expressed as means ± standard deviation (SD). The Shapiro–Wilk test was used on all datasets to assess whether the data were distributed normally. The Pearson’s correlation coefficient was calculated to investigate correlation between RVω and hemodynamic parameters. We evaluated intra-observer and inter-observer reliability in a randomly selected 10 participants. These data were analyzed by two independent readers, blinded to each other’s measurements, and all other data. Intra-observer and inter-observer variability was assessed by repeating the analysis 8 weeks later by the same observer and by a second investigator, respectively. The intraclass correlation coefficient (ICC) and Bland-Altman analysis by calculating the bias (mean difference) and 1.96 SD around the mean difference were assessed. All statistical analyses were carried out using Prism (version 6.0; GraphPad Software, San Diego, CA, USA) and JMP 11 (SAS Institute, Inc., Cary, NC, USA). Statistical significance was defined as a 2-tailed p < 0.05 for all tests.

## Results

### Correlation between MPI and RVω

**Figure 3:**
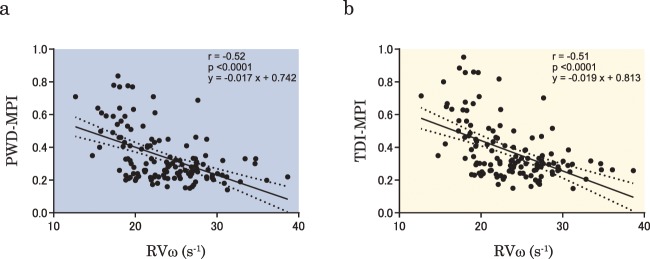
Correlation between the right ventricular pressure angular velocity RVω and the MPI. Relationships are plotted between RVω and PWD-MPI (**a**) and TDI-MPI (**b**). RVω, right ventricular pressure angular velocity; PWD-MPI, myocardial performance index evaluated using pulsed-wave Doppler; TDI-MPI, myocardial performance index evaluated using tissue Doppler imaging.

First, the correlation between the RV MPI and RVω was evaluated to verify the utility of RVω. As shown in Fig. 3, RVω showed significant negative correlations with PWD-MPI (r = −0.52, p < 0.0001) and TDI-MPI (r = −0.51, p < 0.0001) (Fig. 3).

### Correlations between the MPI and other parameters obtained from the RV pressure waveform

**Figure 4:**
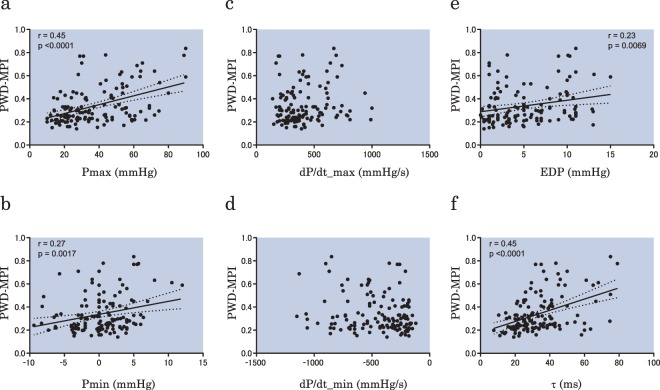
Correlations between PMD-MPI and RV pressure parameters obtained from cardiac catheterization. There are significant correlations between PWD-MPI and Pmax (**a**), Pmin (**b**), EDP (**e**), and τ (**f**), whereas there are no significant correlations with the dP/dt_max (**c**) and dP/dt_min (**d**). PWD-MPI, myocardial performance index evaluated using pulsed-wave Doppler; Pmax, maximum right ventricular pressure; Pmin, minimum right ventricular pressure; dP/dt_max, maximum time derivative of right ventricular pressure; dP/dt_min, minimum time derivative of right ventricular pressure; EDP, right ventricular end-diastolic pressure; τ, time constant of right ventricular relaxation.

**Figure 5:**
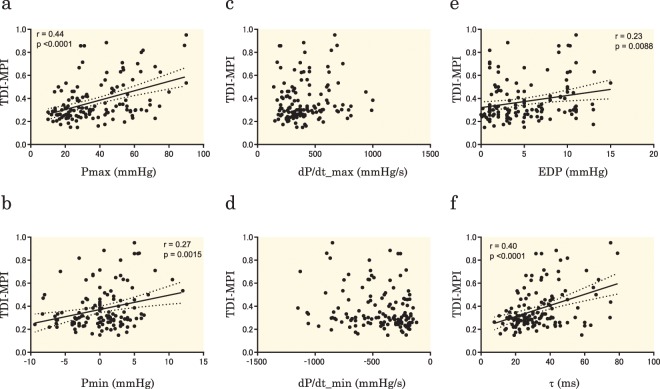
Correlations between TDI-MPI and RV pressure parameters obtained from cardiac catheterization. The TDI-MPI had significant correlations with Pmax (**a**), Pmin (**b**), EDP (**e**), and τ (**f**), whereas no significant correlations are seen with dP/dt_max (**c**) and dP/dt_min (**d**). TDI-MPI, myocardial performance index evaluated using tissue Doppler imaging; Pmax, maximum right ventricular pressure; Pmin, minimum right ventricular pressure; dP/dt_max, maximum time derivative of right ventricular pressure; dP/dt_min, minimum time derivative of right ventricular pressure; EDP, right ventricular end-diastolic pressure; τ, time constant of right ventricular relaxation.

Next, the correlations between the MPI and other RV pressure parameters obtained from cardiac catheterization were assessed. The PWD-MPI had significant correlations with Pmax and Pmin (r = 0.45, p < 0.0001; and r = 0.27, p = 0.0017; respectively) (Fig. 4a,b), whereas there was no correlation between RVω and dP/dt_max or dP/dt_min (Fig. 4c,d). The PWD-MPI had significant correlations with RVEDP and the RV time constant (τ) (r = 0.23, p = 0.0069; and r = 0.45, p < 0.0001; respectively) (Fig. 4e,f). Regarding the TDI-MPI, the results were similar to those of the PWD-MPI. The TDI-MPI had significant correlations with Pmax and Pmin (r = 0.44, p < 0.0001; and r = 0.27, p = 0.0015; respectively) (Fig. 5a,b). The TDI-MPI had no correlation with dP/dt_max or dP/dt_min (Fig. 5c,d). The TDI-MPI had significant positive correlations with RVEDP and the RV time constant (τ) (r = 0.23, p = 0.0088; and r = 0.40, p < 0.0001, respectively) (Fig. 5e,f). From these results, it was seen that RVω had the highest correlation with the MPI among the RVP-derived indices.

### Correlations between RVω and echocardiographic parameters

**Figure 6:**
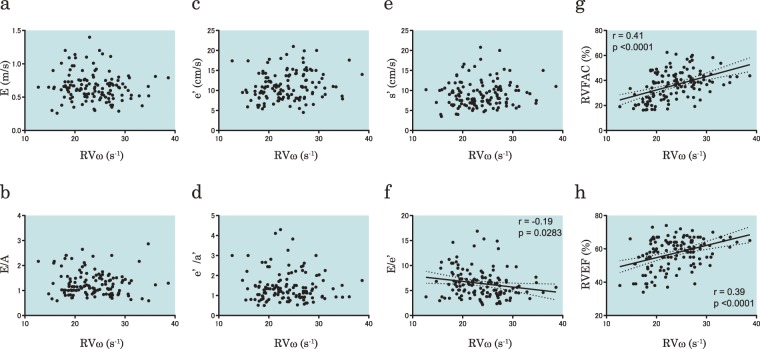
Correlations between RVω and echocardiographic RV parameters. There are no significant correlations between RVω and peak velocity of the tricuspid E wave (**a**), tricuspid E/A (**b**), peak e′ wave velocity of tricuspid annular motion (**c**), e′/a′ of tricuspid annular motion (**d**), and peak s’ wave velocity of tricuspid annular motion (**e**), whereas there are significant correlations with the tricuspid E/e′ (**f**), RVFAC (**g**), and RVEF (**h**). RVω, right ventricular pressure angular velocity; RVFAC, right ventricular fractional area change; RVEF, right ventricular ejection fraction.

In order to elucidate the properties of RVω, the correlations between RVω and RV functional parameters were assessed (Fig. 6). There was a significant negative correlation between RVω and E/e′ (r = −0.19, p = 0.0283). Furthermore, RVω had significant correlations with RVFAC and RVEF (r = 0.41, p < 0.0001, and r = 0.39, p < 0.0001, respectively). RVω did not have significant correlations with the other parameters, including the peak velocity of the E wave, the E/A ratio, peak e′ wave velocity, peak s’ wave velocity, or the e′/a′ ratio. Therefore, it was shown that the PWD-MPI and TDI-MPI had higher correlations with RVω than the other echocardiographic RV parameters.

### Reproducibility

To assess the reproducibilities of PWD-MPI, TDI-MPI, and RVω, intra-observer and inter-observer variabilities were confirmed in 20 randomly selected participants by intraclass correlation coefficient (ICC) and Bland–Altman analysis. RVω had a higher ICC than the PWD-MPI and the TDI-MPI in terms of both intra-observer and inter-observer variabilities (Table 2). RVω measurements proved to be highly reproducible. Bland–Altman analysis also showed minimal bias and substantial agreement for reproducibility.

**Table 2:** Inter- and intra-observer reproducibilities.

Parameter	Intra-observer variability	ICC (Intra-observer)	Inter-observer variability	ICC (Inter-observer)
PWD-MPI	−0.015 ± 0.041	0.921	−0.018 ± 0.041	0.882
TDI-MPI	0.013 ± 0.055	0.959	−0.014 ± 0.061	0.939
RVω (rad/s)	0.114 ± 0.632	0.998	0.125 ± 0.872	0.997

Inter- and intra-observer variabilities (bias ± 1.96 SD [95% limit of agreement]) and the intraclass correlation coefficient (ICC) are shown.

PWD-MPI, pulsed wave Doppler derived myocardial performance index; TDI-MPI, tissue Doppler imaging-derived myocardial performance index; RVω, the novel kinematic model index of the right ventricle, right ventricular pressure angular velocity.

## Discussion

In the present study, the kinematic harmonic oscillation-derived algebraic expression for the angular velocity (ω) obtained using the PPP was applied to the novel analogous MPI index that quantifies ventricular properties. It was demonstrated that the novel index, RVω, had significant negative correlations with PWD-MPI and TDI-MPI. MPI is the time-phase index, which is defined as the sum of isovolumetric contraction (ICT) and relaxation time (IRT) divided by the ejection time, and it indicates the agility of both systolic and diastolic performance corrected by cardiac cycle time^1–3^. On the other hand, RVω is also the time-phase index that represents the angular velocity showing the speed of systolic and diastolic performance. It was concluded that RVω can be an alternative to the MPI and be a useful index to determine the severity of cardiac disease, predict prognosis, and assess the effectiveness of therapy.

Among the RV pressure-based parameters, Pmax, Pmin, RVEDP, the time constant, and RVω had significant correlations with the MPI. RVω had the highest correlation coefficient in its correlations with PWD-MPI and TDI-MPI. In the previous LV MPI studies, it was reported that both dP/dt max and dP/dt min were significantly negatively correlated with MPI^3,23^. These previous investigations indicated that the systolic and diastolic performance represented by dP/dt_max and dP/dt_min had a strong influence on the MPI value^3,23^. However, in the present RV study, dP/dt_max and dP/dt_min were not significantly correlated with the MPI. Instead, both PWD-MPI and TDI-MPI showed significant positive correlations with Pmax, Pmin, RVEDP, and the time constant. These results indicate that higher Pmax and RV pressure overload induce RV myocardial damage and poor performance, which results in a higher MPI^5,6,8–10^. The higher Pmin shows the lower elastic recoil during isovolumic relaxation^24–26^, and it also induces a higher MPI. Higher dP/dt_max and dP/dt_min can generally result from good myocardial performance. On the other hand, higher dP/dt_max and dP/dt_min can also be induced by higher RV systolic pressure overload^26–28^, which can worsen myocardial performance. As a consequence, a higher dP/dt has two significant impacts, positive and negative, on the MPI value. We considered, therefore, that dP/dt_max and dP/dt_min did not have significant correlations with the MPI in the present study. With respect to diastolic function, RVEDP and the time constant are increased with diastolic dysfunction. The present results that RVEDP and the time constant had significant correlations with the MPI are naturally expected, as shown in previous reports^3,23^.

To assess the properties of RVω, the correlations between RVω and RV functional parameters were evaluated. RVω had significant correlations with RVFAC, RVEF, and E/e′. These results indicate that RVω is significantly related to RV systolic and diastolic function.

The RV MPI shows global RV performance as the active energy cycles of contraction and relaxation during ICT and IRT. In harmonic oscillation, the equation of motion can be obtained by Newton’s second law and Hooke’s law, and the restoring force *F* is proportional to the displacement from its equilibrium position x: *F* = −kx = −ω^2^x, where k is a spring constant, and ω is angular velocity. The potential energy stored in a simple harmonic oscillator *U* is shown by the equation *U* = 1/2·kx^2^ = 1/2·ω^2^x^2^. The restoring force and potential energy can be expressed as −ω^2^P, and 1/2·ω^2^P^2^, respectively, by replacing displacement x with pressure P. These equations show that RV systolic and diastolic force and energy are expressed by RVω and RV pressure, and are compatible with the present study result that RVω is related to RV MPI.

Although previous investigations support the clinical significance of the MPI for the monitoring of RV dysfunction^5,6,8–10,29^, the MPI has several limitations. It has been reported that the RV MPI shows pseudonormalization in patients with severe RV infarction^14^, which is accompanied by significant ICT shortening and approximate equalization of end-diastolic RV and PA pressures. Additionally, in patients with surgically corrected tetralogy of Fallot, the noncompliant RV may shorten the RV IRT, resulting in a paradoxically low RV MPI. This may reduce the sensitivity of this index in recognizing patients with RV dysfunction following corrective surgery for tetralogy of Fallot^15^. Furthermore, the effects of valve dysfunction on the MPI must be considered when evaluating ventricular function in patients with valve disease^16,17^. In particular, it has been demonstrated that the MPI can be underestimated in the presence of aortic stenosis because of prolongation of the ejection time. The RV MPI has a weakness in measurement reproducibility^30,31^. In tricuspid pulsed-wave Doppler flow, tracing curves can be unclear because of relatively low flow velocities compared with mitral flow. This may impair time-interval measurements potentially resulting in unsatisfactory accuracy. The novel index, RVω, can overcome these problems of the RV MPI.

The MPI was originally proposed for the assessment of LV systolic and diastolic function. It would be necessary to demonstrate the validation of angular velocity ω in the evaluation of LV performance (LVω) in the future. In the present study, the usefulness of RVω was assessed in patients with potential RV dysfunction, because we thought that RVω has more utility and there is more need for it than LVω. It has been reported that dP/dt_max and dP/dt_min have significant correlations with the MPI in the assessment of LV performance^3,23^, whereas dP/dt_max and dP/dt_min of the RV are influenced by widely interspersed RV peak pressure and are potentially independent of myocardial performance. Thus, the usefulness of RVω was investigated before that of LVω.

### Study limitations

We postulated that RVω from the kinematic phase plane complies with a pure harmonic oscillator. However, RVP contours may not be a pure harmonic oscillatory system. Therefore, damped or forced oscillator might be suitable for a more detailed analysis and should be studied in the future. The aim of the present study was to establish the usefulness and generalizability of RVω using data from clinically heterogeneous patients. Thus, the study design did not analyse the relationships between the index RVω and RV dysfunctional severity, including classes of heart failure, exercise tolerance, and prognosis. Further studies involving specific cardiovascular lesions and longer spans are desirable. Since we did not utilize three-dimensional echocardiography or cardiac magnetic resonance imaging, the RV volume assessment including RVEF has limitation. Although heart rate might have great impact on RVω, it remains to be studied in future. Furthermore, it is necessary to evaluate the correlation and the difference between the noninvasive echocardiographic parameters. In addition, since the sample number in the present study was relatively small, further investigations with larger patient populations are necessary to determine whether this index could serve as a clinically useful assessment tool and become the criterion standard for assessing RV function and predicting patients’ prognosis. The clinical value of the index RVω should be investigated with the respect to prognosis and state change by treatment.

## Conclusion

The present study indicates the clinical feasibility and utility of the kinematic model index, RVω, for assessing global RV performance, incorporating both systolic and diastolic function.

## Data Availability

The datasets generated during and analyzed during the current study are available from the corresponding author on reasonable request.
